# Case Report: Intraocular foreign body coexisting for 30 years

**DOI:** 10.3389/fmed.2025.1513423

**Published:** 2025-04-25

**Authors:** Rong Zhu, Chong Wang, Zhensheng Gu

**Affiliations:** ^1^Department of Ophthalmology, Xinhua Hospital, Affiliated to Shanghai Jiao Tong University School of Medicine, Shanghai, China; ^2^Department of Ophthalmology, Shanghai Sixth People's Hospital, Shanghai Jiao Tong University, Shanghai, China

**Keywords:** intraocular foreign bodies, co-existence, endothelium, trauma, IOL biomaterials

## Abstract

**Background:**

The majority of IOFBs remain in the posterior segment and those in the anterior chamber are uncommon. We report a case of IOFBs in the anterior chamber for 30 years without any symptoms.

**Case presentation:**

The case involves a 30-year-old male individual who was told to have an abnormality in the anterior chamber of his left eye during a physical examination. However, the patient has had no any ocular discomfort symptom within the past 30 years. At the patient’s initial visit, the uncorrected visual acuity of the left eye was 40/50, the corneal endothelial surface exhibited multiple linear and curved scratches, and a transparent foreign body approximately 11 mm in length inhabited in the anterior chamber, touching the endothelium with fan-shaped ends, and the anterior chamber without any signs of inflammation. The endothelial cell count was 1,110 cells/mm^2^. Considered the persistent damage to the corneal endothelium caused by the foreign body, as well as the uncertainty regarding the path of entry and the characteristics of the foreign body, we surgically extracted the intraocular foreign body. No sight-threatening postoperative complications were noted.

**Conclusion:**

A detailed history collection, a thorough physical examination and modern imaging techniques are beneficial for finding IOFBs. Asymptomatic anterior chamber foreign bodies may also cause potential corneal endothelium injury, which should be carefully examined and extracted using appropriate surgical methods to avoid iatrogenic injury.

## Background

Ocular trauma can pose a devastating threat to vision or the globe and is often associated with intraocular foreign bodies (IOFBs), which account for 41% of open globe injuries (1). The majority of IOFBs remain in the posterior segment, and those in the anterior chamber are uncommon, accounting for only approximately 15% of all IOFBs (2). IOFB can present with a variety of symptoms and signs, including visual impairment, pain, eye rupture, cataracts, vitreous hemorrhage and endophthalmitis. However, patients may experience only minimal discomfort when a small, high-velocity object penetrates the eyeball (3). Furthermore, small IOFBs composed of inert materials such as glass, stone, aluminum and silver, hat elicit minimal inflammatory or infectious responses may evade detection during initial clinical evaluation and persist intraocularly for decades (4). We present a case of an intraocular foreign body (IOFB) that remained asymptomatically lodged in the anterior chamber for 30 years before being incidentally discovered during routine physical examination. Despite the successful surgical removal of the IOFB, the corneal endothelial damage caused by the foreign body proved irreversible.

## Case presentation

The case involves a 30-year-old male diagnosed with an abnormality in the anterior chamber of his left eye during a routine physical examination. A “fibrous cord within the anterior chamber of the left eye” was found. The patient was subsequently referred to our institution for comprehensive diagnostic workup. To establish the etiology, we obtained a detailed medical history and performed a full spectrum of ophthalmic evaluations. The patient’s birth history was unremarkable, with delivery at full term. Notably, his parents reported observing congenital corneal opacification in the left eye during infancy, though no formal ophthalmologic assessment was pursued at that time. The opacity demonstrated spontaneous regression over time without residual visual impairment. In addition, the patient had no other ocular complaints within the past 30 years, had no history of ocular trauma or surgery, and denied any history of diabetes, immune-related diseases or familial hereditary diseases. This longitudinal stability supports the hypothesis that the intraocular foreign body has likely persisted within the anterior chamber for approximately 30 years.

**Figure 1:**
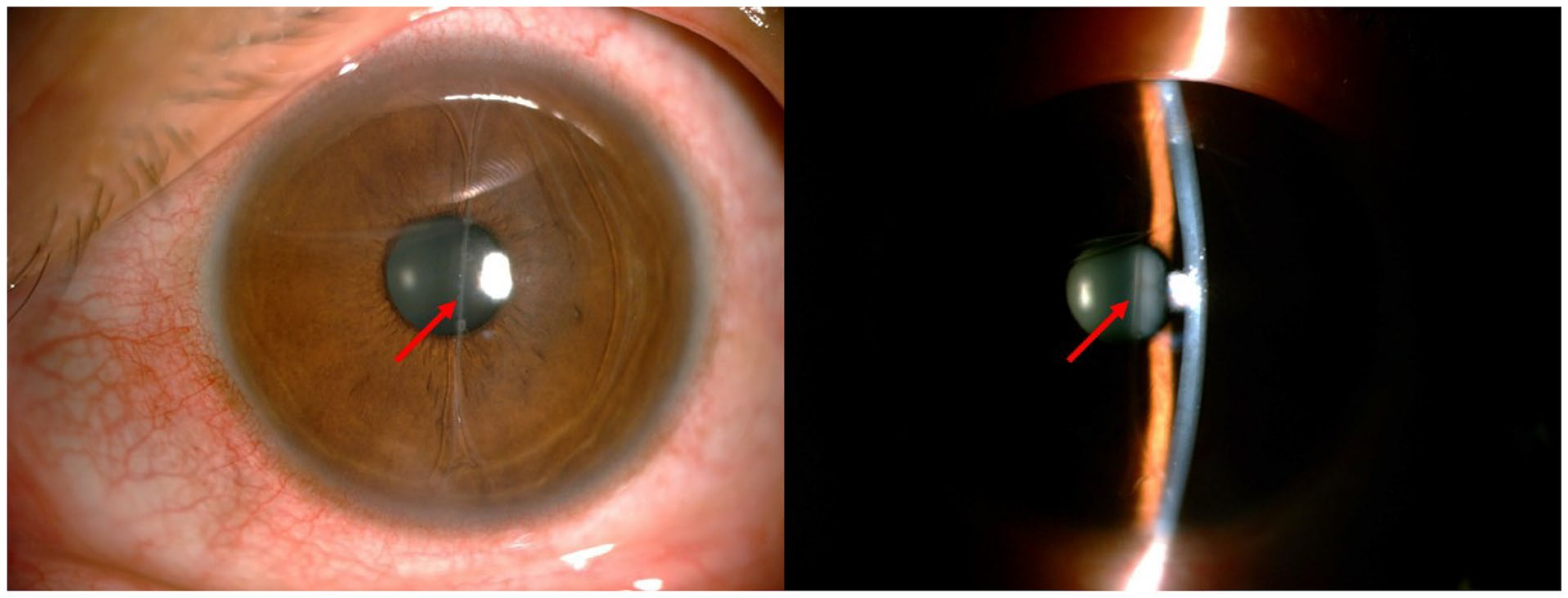
Preoperative photos. The arrow indicates the site of IOFB in the anterior chamber.

**Figure 2:**
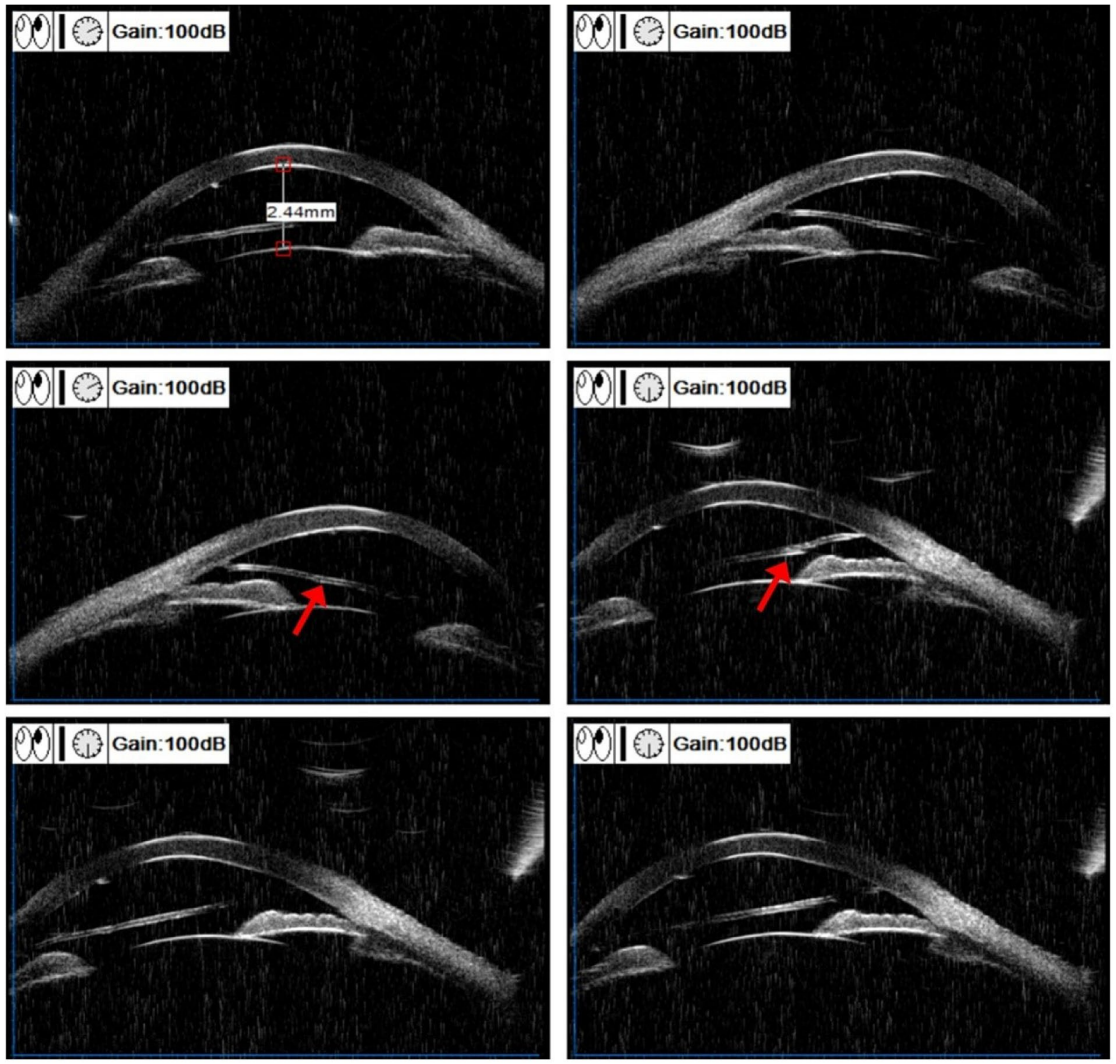
Preoperative UBM. The presence of IOFB (arrow) was in the anterior chamber.

During the initial consultation, comprehensive ophthalmic evaluations were conducted. The left eye demonstrated an uncorrected visual acuity of 40/50 and an intraocular pressure of 16.8 mmHg. Mild conjunctival congestion was observed in the left eye, while the corneal epithelium remained intact without stromal edema or infiltration. Distinctive findings included multiple linear and curvilinear abrasions on the corneal endothelial surface, accompanied by an 11-mm translucent foreign body in the anterior chamber. This foreign body exhibited fan-shaped terminal extensions that maintained contact with the endothelium (Figure 1). The anterior chamber depth was within the normal range without any signs of inflammation. The iris texture was clear, with no evidence of atrophy or synechiae. The pupil had a diameter of 3 mm and was sensitive to light. The lens appeared transparent. There was no obvious opacity in the vitreous. The optic disc showed light red coloration and had pronounced boundaries. The cup-to-disc ratio was 0.4, the arteriovenous ratio was 2/3, the retina was flat, and the foveal reflection was clear. Ultrasound biomicroscopy (UBM) revealed a longitudinal linear echo within the anterior chamber of the left eye. The echo was highly pronounced at both ends and was low in the center. The two ends of the foreign body attached to the corneal endothelium, resulting in a damaged area that appeared less smooth. However, the anterior chamber angle remained open (Figure 2). Additionally, the anterior chamber displayed no significant abnormalities in the orbital magnetic resonance imaging (MRI) scan. The endothelial cell count was 1,110 cells/mm^2^. Studies have shown that when the corneal endothelium is severely damaged by trauma, infection, inflammation or inherited disease or when the endothelial cell count is less than 500 cells/mm^2^, the net pumping capacity can no longer compensate for passive leakage, and corneal edema can develop (5). Given the progressive endothelial trauma from the foreign body’s mechanical contact, combined with indeterminate entry mechanism and material composition, surgical extraction was determined to be clinically warranted following thorough patient consultation.

**Figure 3:**
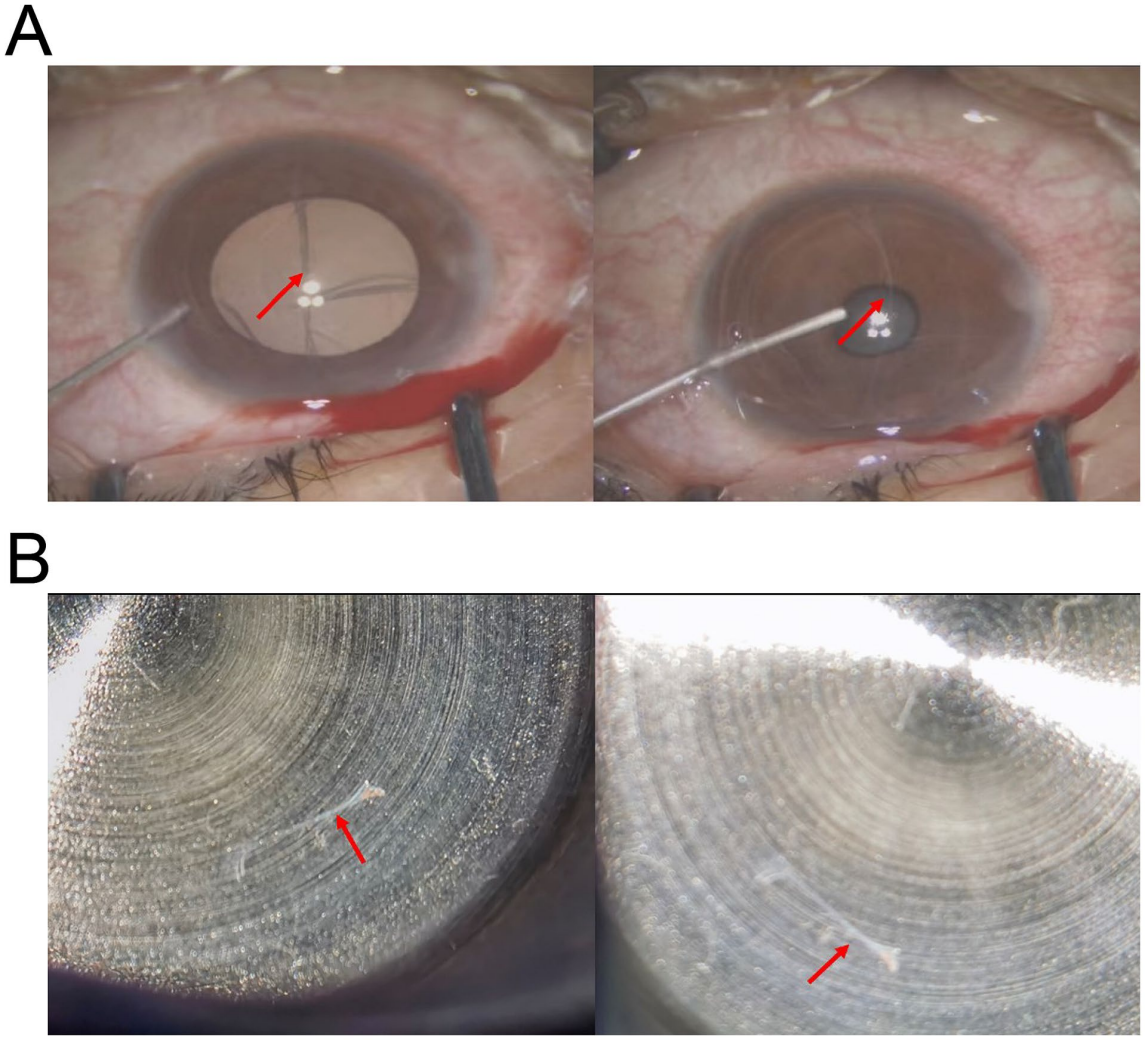
Intraoperative photos in surgeon’s view. **(A)** The photograph shows that the injection of viscoelastic into the anterior chamber caused the IOFB (arrow) to deform. **(B)** The transparent foreign body was completely removed.

**Figure 4:**
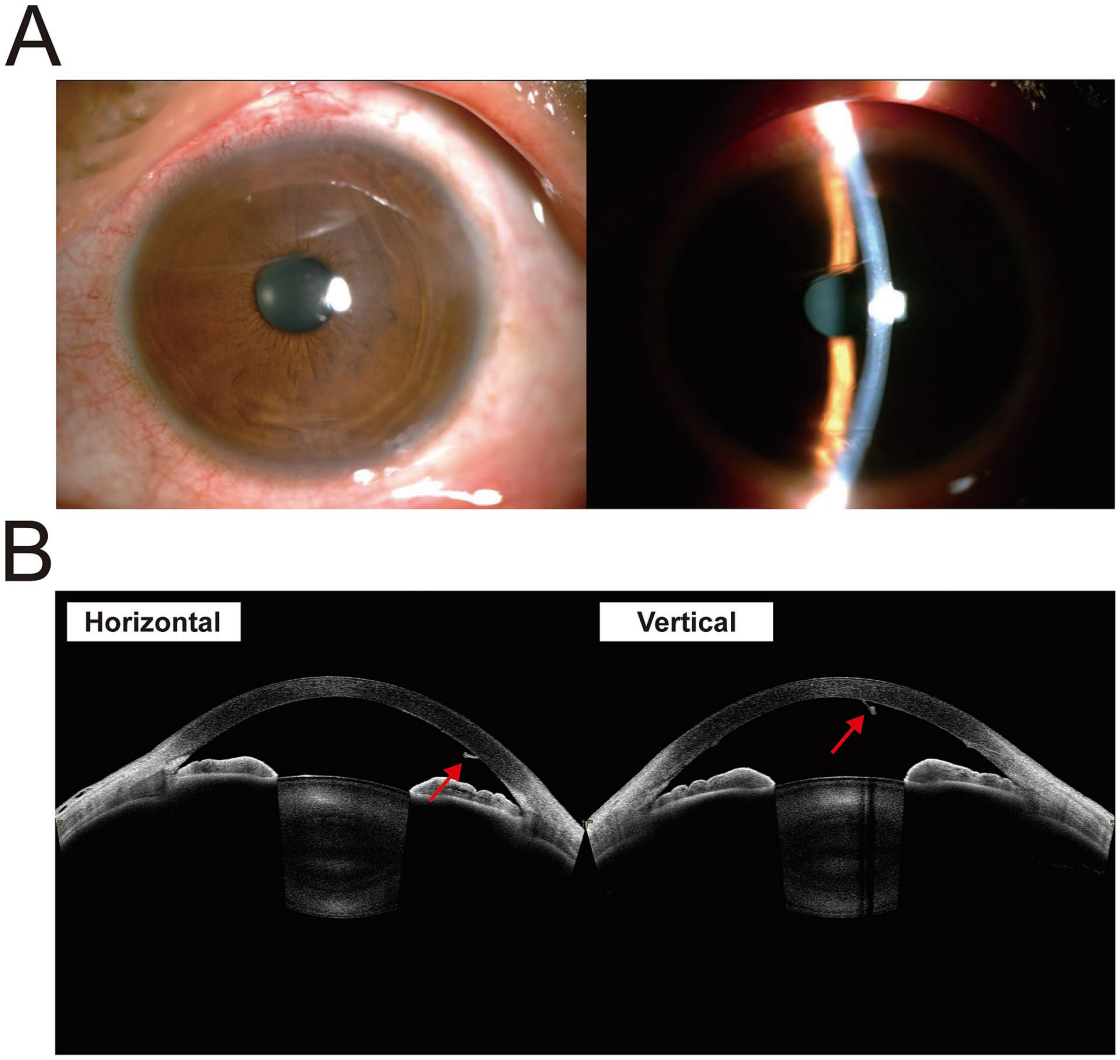
Postoperative photos. **(A)** The slit-lamp photograph shows no inflammatory reaction in the anterior chamber. **(B)** The AS-OCT shows a minor degree of corneal endothelium exfoliation (arrow).

We administered retrobulbar anesthesia to the patient’s left eye. Under the microscope, the foreign body was visible within the left anterior chamber with one end at 6:00 and the other at 12:00, and the ends of the foreign body expanded and adhered to the endothelium. We made a 3 mm wide incision at 11:00 on the superior nasal side of the limbus and slowly injected viscoelastic agents into the anterior chamber to protect the endothelium. During the procedure, we observed the IOFB bending toward the nasal and lens sides (Figure 3A). Due to the tight attachment of the foreign body to the endothelium at both ends, we conducted a blunt separation procedure utilizing viscoelastic agents and a needle. Finally, the IOFB was successfully extracted from the corneal incision using intraocular forceps (Figure 3B). Postsurgical pathological analysis confirmed that the object found in the eye was not a natural biological tissue. After the operation, levofloxacin eye drops were routinely administered for antimicrobial treatment. On the first postoperative day, the slit-lamp examination was performed (Figure 4A). The result showed the conjunctiva was slightly congested. The cornea was transparent. Some scratches were visible on the endothelial surface. There were no inflammatory reactions in the anterior chamber. The pupil had a diameter of 3 mm and was sensitive to light. The lens was transparent. One week after surgery, the endothelial cell count of the operated eye was 1,030 cells/mm^2^. By the end of the sixth month post operation, the uncorrected visual acuity of the left eye was 50/50, and the endothelial cell count had slightly decreased to 968 cells/mm^2^. An examination of the visual field was normal. The visual evoked potential results showed that the latency of the P100 wave was normal in both eyes, and the amplitude of the P100 wave decreased moderately in the right eye and slightly in the left eye. After 1 year follow-up, although the anterior segment optical coherence tomography (AS-OCT) showed a minor degree of corneal endothelium exfoliation, we did not observe further decrease of endothelial cell and the cornea remained transparent (Figure 4B). Subsequently, the patient was advised to maintain regular follow-up appointments to monitor endothelial cell counts and to consider corneal endothelial transplantation in the event of further decline. The whole course of the patient was showed in Timeline (Supplementary Figure 1).

## Discussion

The acquisition of a comprehensive medical history during initial evaluation remains paramount in identifying IOFBs, classically associated with occupational metal fragment injuries from hammering or chiseling activities. However, this patient explicitly denied both occupational exposure to such high-risk tasks and any prior traumatic incidents. Notably, the absence of characteristic symptoms including ocular pain or visual impairment further complicated the determination of injury chronology, mechanism, and foreign body composition. Therefore, comprehensive ophthalmic examinations are necessary to diagnose foreign bodies, and accurate localization and size assessment of IOFBs are also essential aspects of surgical planning to reduce the risk of complications during the perioperative period. There are many tools available to accurately detect and locate IOFBs, including ultrasonography (US), optical coherence tomography (OCT), UBM, computed tomography (CT) and magnetic resonance imaging (MRI), which are critical for diagnosis and surgical planning. CT is often used to detect metallic and nonmetallic foreign bodies, fractures and other soft tissue injuries and is the preferred method for detecting the initial location of foreign bodies. Nonmetallic objects that are difficult to show on CT can be revealed by MRI. However, MRI is strictly contraindicated when ferromagnetic foreign bodies are suspected due to potential magnetic displacement risks (6). UBM is a safe, noninvasive examination and is a useful adjunct to CT and US for the detection and localization of small superficial and intraocular foreign bodies. It may be useful if the presence of a foreign body is suspected and other imaging findings are negative or if a first-line examination reveals a small, nonmetallic and anteriorly located foreign body (7).

The patient in this case underwent UBM and orbital MRI examinations before surgery to determine the foreign body’s material properties (confirmed as nonmagnetic), size, position relationship (between the foreign body and the cornea/iris), and the extent of damage to surrounding tissues. These objective tests guided the surgical planning. The pathway by which the IOFB entered the eye and the nature of the IOFB itself remain the most controversial aspects of this case. The presence of foreign bodies in the anterior chamber for 30 years without subjective symptoms of discomfort or obvious signs of inflammation indicated that the foreign bodies possess the following characteristics: they are nontoxic, noninflammatory, nonantigenic, and noncarcinogenic, exhibit high light transmittance, demonstrate stable physicochemical properties, resist biodegradation, and maintain excellent tissue biocompatibility. The IOFB in the anterior chamber was not visualized on preoperative MRI scans, as its signal intensity matched that of both aqueous humor and vitreous humor. This finding suggested the foreign body was composed of a non-magnetic, highly hydrophilic material. During surgical intervention, intraoperative manipulation revealed that the IOFB exhibited deformability when subjected to viscoelastic agent injection into the anterior chamber, indicating that the foreign body was soft in nature, light in weight and easy to manipulate. Although we are very interested in elucidating the histopathology and chemistry of IOFB, the technical limitations precluded comprehensive analysis in this case. The primary challenges stemmed from two critical factors: First, the delicate nature and diminutive size of the IOFB resulted in fragmentation when we attempted to clamp it for observation and testing, compromising structural integrity for detailed morphological assessment. Second, institutional constraints in specialized ophthalmic pathology services of our hospital limited our capacity to perform high-resolution microscopic examination and energy-dispersive X-ray spectroscopy (EDX) analysis. In addition, standard histological processing protocols proved unsuitable for characterizing the fragmented translucent particulate matter, necessitating specialized micro-analytical techniques currently unavailable at our facility. Regrettably, these combined limitations precluded definitive determination of the histochemical and chemical characteristics of the foreign material. Based on the patient’s history, preoperative examination, and intraoperative findings, we speculated that the properties of the aforementioned IOFB were similar to those of materials commonly used in intraocular lens manufacturing, The primary materials utilized for intraocular lenses include polymethyl methacrylate (PMMA), siloxanes, hydrophilic polyacrylates, and hydrophobic polyacrylates (8). We speculate that the medical auxiliary instruments utilized during delivery might have contained substances capable of causing injury to the patient’s left eye. The foreign body penetrated the eye, inducing mild corneal inflammation. Since the corneal penetrating wound was small and sharply defined, it underwent spontaneous closure shortly after the injury. Although prompt treatment was not administered, the chemically inert nature of the IOFB prevented serious complications. The corneal opacity gradually resolved with time. Unfortunately, the IOFB was located in the anterior chamber, and its movement may have caused damage to the corneal endothelium. Irregular scratches were observed on the patient’s corneal endothelium in a horizontal direction, likely caused by the foreign body entering the eye horizontally. During infancy, when the IOFB exceeded the anterior chamber diameter in length, it became bent and exhibited horizontal movement within the chamber. This motion resulted in partial endothelial abrasions aligned with the direction of displacement. With ocular growth during aging, the eyeball gradually enlarged, the foreign body eventually moves in a vertical direction, extending and fixing. Consequently, despite long-term IOFB retention, the patient’s corneal endothelium maintained a compensatory state with preserved corneal transparency until the time of surgical intervention.

According to the analysis of previous reports, the occurrence of serious complications from an IOFB is related to factors such as its composition, intraocular location, and dimensions. Metal and plant foreign bodies are more likely to cause intraocular infection and inflammation. Iron foreign bodies, in particular, undergo oxidation within the eye and can lead to siderosis, which directly damages the retina and optic nerve (9, 10). In the case of an IOFB that is mobile in the vitreous cavity or a non-encapsulated IOFB in the retina, prompt removal is advisable (11). However, if electroretinography (ERG) shows no signs of ocular siderosis, surgery can be delayed if the IOFB is subretinal or inside a clear lens (10). These patients can be monitored through visual acuity assessments, ophthalmic evaluations, and ERG. Inert materials such as glass and plastic rarely provoke chemical reactions or infections and may remain asymptomatic in the eye for extended periods (2, 12). For chronic, asymptomatic inert foreign bodies, discussing all treatment options with the patient is critical. If the patient prefers to avoid surgery, regular follow-up is essential. Conversely, if poor follow-up compliance is anticipated, surgical removal of the IOFB is recommended.

In conclusion, IOFBs may be missed for many years if a small and self-healing penetrating wound is not accompanied by signs of inflammation or infection. This case is a reminder that a detailed history collection, a thorough physical examination and modern imaging techniques are beneficial for identifying IOFBs. Asymptomatic anterior chamber foreign bodies may also cause potential corneal endothelium injury and should be carefully examined and extracted using appropriate surgical methods to avoid iatrogenic injury. Furthermore, the distinct characteristics of the foreign body in this case suggest its potential as a new, ideal intraocular implant material. Our future research will continue to investigate its properties, with the aim of discovering a new intraocular lens material suitable for human eyes.

## Data Availability

The raw data supporting the conclusions of this article will be made available by the authors, without undue reservation.
