# Supplementary figures and images for: Case Report: Intraocular foreign body coexisting for 30 years
Source: Front Med (Lausanne). 2025 Apr 25;12:1513423. doi: 10.3389/fmed.2025.1513423 (PMC12063503; doi:10.3389/fmed.2025.1513423)

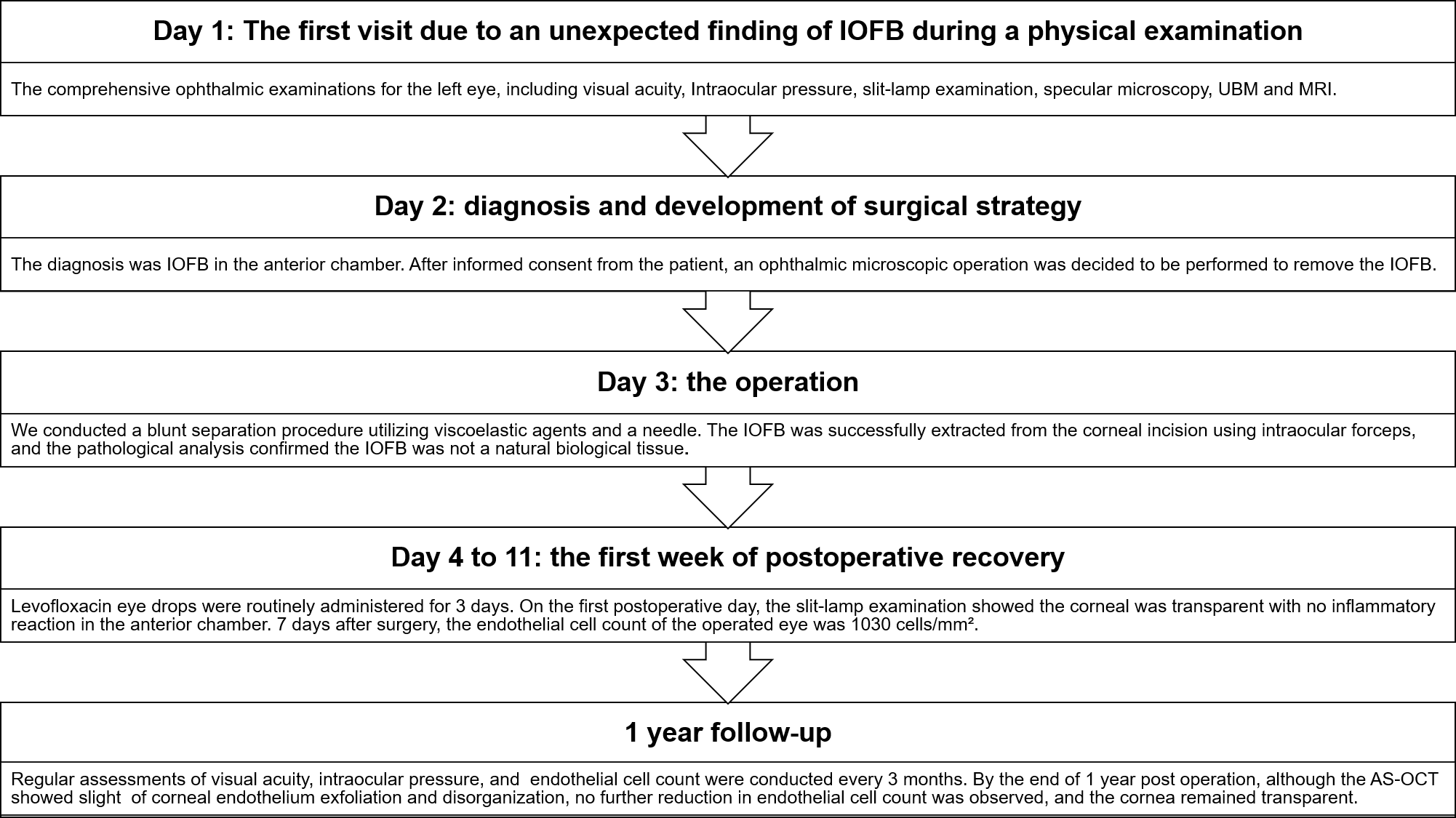

Supplement: Supplementary file 1 [file Image_1.tif]
